# Assessment of heavy metal and radioactive element levels in the ovarian follicular fluid of pregnant and non-pregnant Kyrgyz breed mares

**DOI:** 10.5455/javar.2024.k808

**Published:** 2024-09-29

**Authors:** Ali Risvanli, Fatih Hatipoglu, Ruslan Salykov, Necati Timurkaan, Nariste Kadiralieva, Kaliyman K. Kasymalieva, Ibrahim Seker, Kobil Jurakulov, Nuriddin Ruzikulov, Abdugani Abdurasulov, Cahit Kalkan

**Affiliations:** 1 Kyrgyzstan-Turkey Manas University Faculty of Veterinary Medicine, Bishkek, Kyrgyzstan; 2 Faculty of Veterinary Medicine, Firat University, Elazığ, Türkiye; 3 Faculty of Veterinary Medicine, Selcuk University, Konya, Türkiye; 4 Livestock and Biotechnology, Samarkand State University of Veterinary Medicine, Samarkand, Uzbekistan; 5 Faculty of Natural Science, Tourism and Agricultural Technology, Osh State University, Osh, Kyrgyzstan

**Keywords:** Kyrgyz mare, ovarian follicular fluid, heavy metal, radioactive element

## Abstract

**Objective:**

In the current study, we aimed to ascertain the levels of heavy metals and radioactive elements in the ovarian follicular fluids of pregnant and non-pregnant Kyrgyz mares.

**Materials and Methods:**

To this end, follicular fluids were obtained from 49 Kyrgyz mares aged 3–5 years. The mares were in various stages of pregnancy (Group 1, *n = *27) or were non-pregnant (Group 2, *n = *22). We measured the levels of cadmium (Cd), lead (Pb), mercury (Hg), antimony (Sb), cobalt (Co), potassium (K), uranium (U), nickel (Ni), and thorium (Th) in follicular fluids using atomic emission mass spectrometry. Subsequently, the data were statistically analyzed according to whether the mare was pregnant or not, the pregnancy stage, the presence or absence of the corpus luteum in the ovaries, the corpus luteum’s diameter if present, and the presence or absence and diameter of follicles.

**Results:**

We found that K levels were higher in non-pregnant mares (0.00564 ± 0.000590 ppm, *p* = 0.009). Furthermore, Ni (0.01033 ± 0.001880 ppm, *p* = 0.07) and K (0.00563 ± 0.000498 ppm, *p* = 0.026) levels were higher in mares with a follicle diameter of 1–3 cm. However, these results did not change according to the month of pregnancy, number of follicles, presence or absence of the corpus luteum in the ovaries, number of corpora lutea, and their diameters.

**Conclusion:**

Thus, we concluded that heavy metal and radioactive element levels in the ovarian follicular fluids of Kyrgyz mares do not significantly change with pregnancy status, and there are limited detrimental effects of pollution on the follicular fluids.

## Introduction

Environmental factors, including heavy metals, are known to have detrimental effects on reproductive health. In industrialized nations, exposure to heavy metal pollution—a consequence of environmental contamination—has been suggested as a contributing factor to rising infertility rates in women. Accumulation of heavy metals in the body may lead to infertility by disrupting the hypothalamic-pituitary-ovarian axis, impairing the production of hormones such as estradiol or progesterone, or causing chromosomal damage [1]. The detection of heavy metals in the genital organs of both men and women highlights the potential reproductive toxicity of these substances [2]. Heavy metals such as arsenic, cadmium, and lead are particularly concerning, as they are believed to increase oxidative stress, trigger cellular apoptosis, disrupt endocrine function, and induce epigenetic changes [3].

Pb and Cd are two of the most recognized reproductive toxins, often encountered through environmental exposure. These metals have been shown to have harmful effects on chromatin integrity and are prevalent in the environment, leading to their gradual accumulation in the body [4]. The oxidative stress induced by heavy metals can cause significant damage to body cells, particularly to lipid membranes. Gametes are especially vulnerable to oxidative stress, likely due to the weakening of cellular defense mechanisms [5,6].

Free radical processes are among the numerous mechanisms still under investigation regarding heavy metal toxicity. Human exposure to Pb and Cd often coincides with exposure to Zn, a metal that, in small amounts, can have beneficial effects on male fertility and potentially counteract the adverse effects of Cd, Pb, and other metals. Essential metals such as Cu, Zn, and Se play crucial roles in reproductive health as components of many enzymes. However, at elevated levels, these metals can also be harmful [7]. Pb, for instance, can antagonize or compete with Cu, Zn, and Se, inhibiting cellular functions and impairing antioxidant defenses [8]. The toxic effects of Cd are thought to be partly due to its classification as a metalloestrogen—an inorganic metal ion that binds to and activates estrogen receptors [9]. Martino et al. [10] reported that even nanomolar levels of Cd can negatively affect oocyte fertilization by inducing oxidative damage in the cumulus oophorus.

Ni exposure is associated with various health conditions, including bronchitis, lung cancer, alveolar inflammation, and reproductive system disorders. Ni exposure also promotes the production of reactive oxygen species (ROS), causes errors in DNA replication, and activates signal transduction pathways [11]. Additionally, Ni increases the concentrations of fllicle-stimulating hormone and LH, causes ovarian lymphocytosis due to vasodilation, and leads to cavitation and enlargement of luteal cells [12]. Moreover, Ni can induce teratogenic effects by altering placental permeability and reducing its function [13,14].

Hg is another heavy metal that induces oxidative stress, leading to male reproductive dysfunction. Hg disrupts the endocrine system in testicular cells by promoting an imbalance between prooxidants and antioxidants. Even at low levels, Hg exposure can cause severe reproductive complications such as stillbirth, spontaneous abortion, fetal malformations, and infertility [15].

Depleted U, which is left after the enrichment process, emits alpha and beta particles, making it more permeable and capable of altering DNA methylation. U toxicity can also trigger ROS production, hormonal imbalances, gene expression disruptions, and inflammation [16,17].

K levels, like those of other electrolytes, play a significant role in both male and female fertility. Both deficient and excessive levels of K can lead to infertility [1,13].

The aim of this study was to determine the levels of heavy metals and radioactive elements in the ovarian follicular fluids of pregnant and non-pregnant Kyrgyz breed mares. This research seeks to assess the extent of environmental pollution in critical environments, such as follicular fluid, to better understand its impact on reproductive health.

## Materials and Methods

The project was conducted from August to September 2024, involving 49 Kyrgyz mares aged between 3 and 5 years, all of which were slaughtered in a Bishkek, Kyrgyzstan, abattoir. Approval was duly obtained from the KTMU Experimental Animals Ethics Committee (17/11/2023-2023/05). The animals selected for the study were clinically healthy mares that had lived in and around Bishkek since birth.

The mares used for the study were randomly divided into two groups. The groups were constituted as follows: Group 1 (*n = *27): Mares at different stages of pregnancy; Group 2 (*n = *22): It was formed from non-pregnant mares.

Once the ovaries were transported to the laboratory in an ice-filled container, the diameters of the follicles and the corpus luteum were examined with calipers. Any follicles and corpus luteum exhibiting cystic or other pathological conditions were excluded from evaluation.

### Collection of follicle fluids

Follicular fluids were aspirated from the follicles using 19-gauge needles. These obtained follicular fluids were subsequently stored in a deep freezer at −80°C until the measurements were performed. Cd, Pb, Hg, Sb, Co, K, U, Ni, and Th were measured in 49 follicular fluids obtained from mares.

### Measurements in follicle fluid

Cd, Pb, Hg, Sb, Co, K, U, Ni, and Th were measured using atomic emission mass spectrometry. The measurements were conducted at the Stewart Assay and Environmental Laboratories in Kara-Balta, Kyrgyzstan.

A 10 μl sample was digested using repeated additions of 0.5% HNO₃ at a controlled drying temperature, followed by treatment with H₂O₂. Hot digestion was then performed, and the resulting solution was filtered through filter paper. The final volume of the solution was adjusted using bidistilled water.

For the quantification of Pb and Cd, the wavelengths used were 283.3 and 228.8 nm, respectively. The detection limits for Pb and Cd were 0.045 and 0.002 mg/kg. The concentrations in the standard solutions used for Pb and Cd analysis were 100 ± 0.01 and 100 ± 0.02 mg/kg, respectively.

The data were evaluated based on whether the mare was pregnant or not. If pregnant, additional considerations included the month of pregnancy, the presence of a corpus luteum in the ovaries, the diameter of the corpus luteum if present, and the presence and size of the largest follicle.

### Statistical analyses

In the study, we first calculated descriptive statistics using data gathered from relevant groups for the parameters analyzed. We then evaluated, for every parameter in each group, whether the data satisfied parametric test assumptions and exhibited a normal distribution. Ultimately, we determined that the data for all examined parameters neither met the parametric test assumptions nor showed a normal distribution.

The Mann-Whitney U test was used for comparisons between two groups, while the Kruskal-Wallis analysis of variance was implemented for comparisons among three groups. A significance level was established at *p *< 0.05 for all statistical analyses. All such analyses were conducted using SPSS 22.0.

## Results

Information regarding the variations in Cd, Pb, Hg, Sb, Co, K, U, Ni, and Th levels in follicle fluids, according to the pregnancy status of mares, is summarized in Table 1. Notably, K levels were found to be higher in non-pregnant mares (0.00564 ± 0.000590 ppm, *P* = 0.009). However, there were no significant differences in Cd, Pb, Hg, Sb, Co, U, Ni, and Th levels.

Information about the fluctuations in Cd, Pb, Hg, Sb, Co, K, U, Ni, and Th levels in follicle fluids corresponding to the pregnancy month of mares is summarized in Table 2. Consequently, it was determined that the results remained stable regardless of the pregnancy month (*p > *0.05).

The changes in the levels of Cd, Pb, Hg, Sb, Co, K, U, Ni, and Th in mare follicle fluids based on follicle numbers are summarized in Table 3. The results revealed no significant change regarding the follicle numbers (*p* > 0.05).

Information regarding the variations in Cd, Pb, Hg, Sb, Co, K, U, Ni, and Th levels in the follicle fluids according to the follicle diameter of mares is summarized in Table 4. Specifically, Ni (0.01033 ± 0.001880 ppm, *p* = 0.07) and K levels were higher in mares with a follicle diameter of 1–3 cm (0.00563 ± 0.000498 ppm, *p* = 0.026). The levels of Cd, Pb, Hg, Sb, Co, U, and Th did not exhibit significant differences (*p > *0.05).

Information on the variations of Cd, Pb, Hg, Sb, Co, K, U, Ni, and Th levels in follicular fluids in relation to the presence of corpus luteum in mare ovaries is summarized in Table 5. Consequently, it was observed that the results did not vary based on the presence of corpus luteum (*p > *0.05).

**Table 1.** Distribution of results according to pregnancy status.

Pregnancy		Cd (ppm)	Hg (ppm)	Pb (ppm)	Sb (ppm)	Co (ppm)	K (ppm)	U (ppm)	Ni (ppm)	Th (ppm)
Pregnant (group 1)	*n*	27	27	27	27	27	27	27	27	27
	Mean	.00300	.00500	.00200	.04274	.00300	.00515	.00515	.00800	.00500
	Std. error of mean	.000000	.000000	.000000	.007520	.000000	.000148	.000148	.001724	.000000
	Median	.00300	.00500	.00200	.02500	.00300	.00500	.00500	.00500	.00500
	Minimum	.003	.005	.002	.009	.003	.005	.005	.005	.005
	Maximum	.003	.005	.002	.159	.003	.009	.009	.049	.005
Non-pregnant (group 2)	*n*	22	22	22	22	22	22	22	22	22
	Mean	.00327	.00500	.00200	.04186	.00300	.00564	.00564	.00841	.00500
	Std. error of mean	.000199	.000000	.000000	.007893	.000000	.000590	.000590	.001262	.000000
	Median	.00300	.00500	.00200	.03150	.00300	.00500	.00500	.00500	.00500
	Minimum	.003	.005	.002	.005	.003	.005	.005	.005	.005
	Maximum	.007	.005	.002	.163	.003	.018	.018	.025	.005
Total	*n*	49	49	49	49	49	49	49	49	49
	Mean	.00312	.00500	.00200	.04235	.00300	.00537	.00537	.00818	.00500
	Std. error of mean	.000091	.000000	.000000	.005396	.000000	.000276	.000276	.001096	.000000
	Median	.00300	.00500	.00200	.02600	.00300	.00500	.00500	.00500	.00500
	Minimum	.003	.005	.002	.005	.003	.005	.005	.005	.005
	Maximum	.007	.005	.002	.163	.003	.018	.018	.049	.005
*p*		-	-	-	-	-	.009	-	-	-

- *p *> 0.05

**Table 2.** Distribution of results according to pregnancy months.

Pregnancy months		Cd (ppm)	Hg (ppm)	Pb (ppm)	Sb (ppm)	Co (ppm)	K (ppm)	U (ppm)	Ni (ppm)	Th (ppm)
1–2	*n*	14	14	14	14	14	14	14	14	14
	Mean	.00300	.00500	.00200	.03671	.00300	.00500	.00500	.00629	.00500
	Std. error of mean	.000000	.000000	.000000	.008409	.000000	.000000	.000000	.000773	.000000
	Median	.00300	.00500	.00200	.02250	.00300	.00500	.00500	.00500	.00500
	Minimum	.003	.005	.002	.009	.003	.005	.005	.005	.005
	Maximum	.003	.005	.002	.118	.003	.005	.005	.013	.005
3–4	*n*	7	7	7	7	7	7	7	7	7
	Mean	.00300	.00500	.00200	.05429	.00300	.00557	.00557	.01157	.00500
	Std. error of mean	.000000	.000000	.000000	.014848	.000000	.000571	.000571	.006244	.000000
	Median	.00300	.00500	.00200	.05600	.00300	.00500	.00500	.00500	.00500
	Minimum	.003	.005	.002	.011	.003	.005	.005	.005	.005
	Maximum	.003	.005	.002	.132	.003	.009	.009	.049	.005
5–6	*n*	6	6	6	6	6	6	6	6	6
	Mean	.00300	.00500	.00200	.04333	.00300	.00500	.00500	.00783	.00500
	Std. error of mean	.000000	.000000	.000000	.023181	.000000	.000000	.000000	.002638	.000000
	Median	.00300	.00500	.00200	.02200	.00300	.00500	.00500	.00500	.00500
	Minimum	.003	.005	.002	.015	.003	.005	.005	.005	.005
	Maximum	.003	.005	.002	.159	.003	.005	.005	.021	.005
Total	*n*	27	27	27	27	27	27	27	27	27
	Mean	.00300	.00500	.00200	.04274	.00300	.00515	.00515	.00800	.00500
	Std. error of mean	.000000	.000000	.000000	.007520	.000000	.000148	.000148	.001724	.000000
	Median	.00300	.00500	.00200	.02500	.00300	.00500	.00500	.00500	.00500
	Minimum	.003	.005	.002	.009	.003	.005	.005	.005	.005
	Maximum	.003	.005	.002	.159	.003	.009	.009	.049	.005
*p*		-	-	-	-	-	-	-	-	-

- *p *> 0.05

The variations in Cd, Pb, Hg, Sb, Co, K, U, Ni, and Th levels in the follicular fluids, according to the number of corpus luteum in mare ovaries, are summarized in Table 6. It was determined that these results did not vary based on the number of corpus luteum (*p > *0.05).

Information regarding the changes in the levels of Cd, Pb, Hg, Sb, Co, K, U, Ni, and Th in follicular fluids in relation to the diameter of the corpus luteum in mare ovaries is summarized in Table 7. According to these findings, there were no significant changes in results based on the diameter of the corpus luteum (*p > *0.05).

## Discussion

Follicular fluid is an ultrafiltrate of blood plasma, selectively excluding high molecular weight proteins through the blood-follicle barrier. It fills the growing follicle, providing a crucial environment for the developing oocyte [18]. The composition of follicular fluid can reflect environmental exposures that impact early reproductive stages, including oocyte and embryo quality. It is hypothesized that follicular fluid provides a more accurate estimate of the biologically effective dose of toxicants affecting the oocyte than other measures [19]. Therefore, managing exposure to toxic elements could be key in treating infertility and improving the success rates of assisted reproductive techniques [3]. Compared to blood and urine samples, follicular fluid offers a more precise reflection of the microenvironment surrounding the developing oocyte, making it a superior biomarker for exposures that could influence reproductive outcomes [20]. However, research by El Mohr et al. [21] found intra-follicular differences in metal levels within the same woman, and Silberstein et al. [22] noted that element concentrations often vary between small and large follicles. In the current study, levels of Ni (0.01033 ± 0.001880 ppm) and K were higher in those with a follicle diameter of 1–3 cm (0.00563 ± 0.000498 ppm). However, no significant variation was observed in the levels of Cd, lead Pb, Hg, Sb, Co, U, or Th.

**Table 3.** Distribution of results according to follicle numbers.

Follicle numbers		Cd (ppm)	Hg (ppm)	Pb (ppm)	Sb (ppm)	Co (ppm)	K (ppm)	U (ppm)	Ni (ppm)	Th (ppm)
1	*n*	31	31	31	31	31	31	31	31	31
	Mean	.00319	.00500	.00200	.04710	.00300	.00555	.00555	.00848	.00500
	Std. error of mean	.000142	.000000	.000000	.007334	.000000	.000435	.000435	.001037	.000000
	Median	.00300	.00500	.00200	.03300	.00300	.00500	.00500	.00500	.00500
	Minimum	.003	.005	.002	.005	.003	.005	.005	.005	.005
	Maximum	.007	.005	.002	.163	.003	.018	.018	.025	.005
2->	*n*	18	18	18	18	18	18	18	18	18
	Mean	.00300	.00500	.00200	.03417	.00300	.00506	.00506	.00767	.00500
	Std. error of mean	.000000	.000000	.000000	.007357	.000000	.000056	.000056	.002436	.000000
	Median	.00300	.00500	.00200	.02250	.00300	.00500	.00500	.00500	.00500
	Minimum	.003	.005	.002	.009	.003	.005	.005	.005	.005
	Maximum	.003	.005	.002	.132	.003	.006	.006	.049	.005
Total	*n*	49	49	49	49	49	49	49	49	49
	Mean	.00312	.00500	.00200	.04235	.00300	.00537	.00537	.00818	.00500
	Std. error of mean	.000091	.000000	.000000	.005396	.000000	.000276	.000276	.001096	.000000
	Median	.00300	.00500	.00200	.02600	.00300	.00500	.00500	.00500	.00500
	Minimum	.003	.005	.002	.005	.003	.005	.005	.005	.005
	Maximum	.007	.005	.002	.163	.003	.018	.018	.049	.005
*p*		-	-	-	-	-	-	-	-	-

- *p *> 0.05

**Table 4.** Evaluation of results according to follicle diameter.

Follicle diameter (cm)		Cd (ppm)	Hg (ppm)	Pb (ppm)	Sb (ppm)	Co (ppm)	K (ppm)	U (ppm)	Ni (ppm)	Th
1-3	*n*	27	27	27	27	27	27	27	27	27
	Mean	.00322	.00500	.00200	.05011	.00300	.00563	.00563	.01033	.00500
	Std. error of mean	.000163	.000000	.000000	.008349	.000000	.000498	.000498	.001880	.000000
	Median	.00300	.00500	.00200	.03300	.00300	.00500	.00500	.00500	.00500
	Minimum	.003	.005	.002	.009	.003	.005	.005	.005	.005
	Maximum	.007	.005	.002	.163	.003	.018	.018	.049	.005
4->	*n*	22	22	22	22	22	22	22	22	22
	Mean	.00300	.00500	.00200	.03282	.00300	.00505	.00505	.00555	.00500
	Std. error of mean	.000000	.000000	.000000	.005872	.000000	.000045	.000045	.000376	.000000
	Median	.00300	.00500	.00200	.02200	.00300	.00500	.00500	.00500	.00500
	Minimum	.003	.005	.002	.005	.003	.005	.005	.005	.005
	Maximum	.003	.005	.002	.118	.003	.006	.006	.013	.005
Total	*n*	49	49	49	49	49	49	49	49	49
	Mean	.00312	.00500	.00200	.04235	.00300	.00537	.00537	.00818	.00500
	Std. error of mean	.000091	.000000	.000000	.005396	.000000	.000276	.000276	.001096	.000000
	Median	.00300	.00500	.00200	.02600	.00300	.00500	.00500	.00500	.00500
	Minimum	.003	.005	.002	.005	.003	.005	.005	.005	.005
	Maximum	.007	.005	.002	.163	.003	.018	.018	.049	.005
	*p*	-	-	-	-	-	.026	-	.007	-

- *p *> 0.05

**Table 5.** Distribution of results according to corpus luteum status.

Corpus luteum		Cd (ppm)	Hg (ppm)	Pb (ppm)	Sb (ppm)	Co (ppm)	K (ppm)	U (ppm)	Ni (ppm)	Th (ppm)
Presence	*n*	40	40	40	40	40	40	40	40	40
	Mean	.00315	.00500	.00200	.04315	.00300	.00543	.00543	.00835	.00500
	Std. error of mean	.000111	.000000	.000000	.006341	.000000	.000338	.000338	.001319	.000000
	Median	.00300	.00500	.00200	.02550	.00300	.00500	.00500	.00500	.00500
	Minimum	.003	.005	.002	.009	.003	.005	.005	.005	.005
	Maximum	.007	.005	.002	.163	.003	.018	.018	.049	.005
Absence	*n*	9	9	9	9	9	9	9	9	9
	Mean	.00300	.00500	.00200	.03878	.00300	.00511	.00511	.00744	.00500
	Std. error of mean	.000000	.000000	.000000	.008841	.000000	.000111	.000111	.001203	.000000
	Median	.00300	.00500	.00200	.03300	.00300	.00500	.00500	.00500	.00500
	Minimum	.003	.005	.002	.005	.003	.005	.005	.005	.005
	Maximum	.003	.005	.002	.074	.003	.006	.006	.014	.005
Total	*n*	49	49	49	49	49	49	49	49	49
	Mean	.00312	.00500	.00200	.04235	.00300	.00537	.00537	.00818	.00500
	Std. error of mean	.000091	.000000	.000000	.005396	.000000	.000276	.000276	.001096	.000000
	Median	.00300	.00500	.00200	.02600	.00300	.00500	.00500	.00500	.00500
	Minimum	.003	.005	.002	.005	.003	.005	.005	.005	.005
	Maximum	.007	.005	.002	.163	.003	.756	.018	.049	.005
	*p*	-	-	-	-	-	-	-	-	-

- *p* > 0.05

**Table 6.** Distribution of results according to the number of corpus luteum.

Corpus luteum		Cd (ppm)	Hg (ppm)	Pb (ppm)	Sb (ppm)	Co (ppm)	K (ppm)	U (ppm)	Ni (ppm)	Th (ppm)
1	*n*	40	40	40	40	40	40	40	40	40
	Mean	.00315	.00500	.00200	.04455	.00300	.00543	.00543	.00835	.00500
	Std. error of mean	.000111	.000000	.000000	.006353	.000000	.000338	.000338	.001319	.000000
	Median	.00300	.00500	.00200	.02800	.00300	.00500	.00500	.00500	.00500
	Minimum	.003	.005	.002	.009	.003	.005	.005	.005	.005
	Maximum	.007	.005	.002	.163	.003	.018	.018	.049	.005
2->	*n*	9	9	9	9	9	9	9	9	9
	Mean	.00300	.00500	.00200	.03256	.00300	.00511	.00511	.00744	.00500
	Std. error of mean	.000000	.000000	.000000	.007879	.000000	.000111	.000111	.001203	.000000
	Median	.00300	.00500	.00200	.02300	.00300	.00500	.00500	.00500	.00500
	Minimum	.003	.005	.002	.005	.003	.005	.005	.005	.005
	Maximum	.003	.005	.002	.068	.003	.006	.006	.014	.005
Total	*n*	49	49	49	49	49	49	49	49	49
	Mean	.00312	.00500	.00200	.04235	.00300	.00537	.00537	.00818	.00500
	Std. error of mean	.000091	.000000	.000000	.005396	.000000	.000276	.000276	.001096	.000000
	Median	.00300	.00500	.00200	.02600	.00300	.00500	.00500	.00500	.00500
	Minimum	.003	.005	.002	.005	.003	.005	.005	.005	.005
	Maximum	.007	.005	.002	.163	.003	.018	.018	.049	.005
*p*		-	-	-	-	-	-	-	-	-

- *p* > 0.05

**Table 7.** Distribution of results according to corpus luteum diameter.

Corpus luteum diameter (cm)		Cd (ppm)	Hg (ppm)	Pb (ppm)	Sb (ppm)	Co (ppm)	K (ppm)	U (ppm)	Ni (ppm)	Th (ppm)
1–3	*n*	15	15	15	15	15	15	15	15	15
	Mean	.00300	.00500	.00200	.03773	.00300	.00527	.00527	.00653	.00500
	Std. Error of Mean	.000000	.000000	.000000	.009738	.000000	.000267	.000267	.001055	.000000
	Median	.00300	.00500	.00200	.02100	.00300	.00500	.00500	.00500	.00500
	Minimum	.003	.005	.002	.009	.003	.005	.005	.005	.005
	Maximum	.003	.005	.002	.159	.003	.009	.009	.021	.005
4->	*n*	25	25	25	25	25	25	25	25	25
	Mean	.00324	.00500	.00200	.04640	.00300	.00552	.00552	.00944	.00500
	Std. Error of Mean	.000176	.000000	.000000	.008377	.000000	.000520	.000520	.002000	.000000
	Median	.00300	.00500	.00200	.02600	.00300	.00500	.00500	.00500	.00500
	Minimum	.003	.005	.002	.009	.003	.005	.005	.005	.005
	Maximum	.007	.005	.002	.163	.003	.018	.018	.049	.005
Total	*n*	40	40	40	40	40	40	40	40	40
	Mean	.00315	.00500	.00200	.04315	.00300	.00543	.00543	.00835	.00500
	Std. Error of Mean	.000111	.000000	.000000	.006341	.000000	.000338	.000338	.001319	.000000
	Median	.00300	.00500	.00200	.02550	.00300	.00500	.00500	.00500	.00500
	Minimum	.003	.005	.002	.009	.003	.005	.005	.005	.005
	Maximum	.007	.005	.002	.163	.003	.018	.018	.049	.005
	*p*	-	-	-	-	-	-	-	-	-

- *p *> 0.05

Toxic metals can accumulate in follicular fluid, leading to damage in granulosa cells, impaired hormone synthesis, and reduced oocyte quality. This can result in pregnancy loss or premature calving. Moreover, these metals can cross the placenta, potentially causing developmental issues in the fetus [23]. For example, the accumulation of Pb in cow’s follicular fluid has been found to significantly inhibit folliculogenesis [24]. A study on mice suggests that a dose of 10 mg/kg of Pb over 15 weeks can impair folliculogenesis and increase the number of atretic primary follicles [25]. Although metals such as As, Cd, Mg, and Zn have been detected in human follicular fluid, no association with pregnancy rates has been reported [26]. Interestingly, Silberstein et al. [27] found that Pb concentrations in follicular fluid can exceed blood levels and are inversely associated with pregnancy outcomes. In this study, no differences were observed in the levels of Cd, Pb, Hg, Sb, Co, U, Ni, or Th, regardless of the mares’ pregnancy status. The discrepancies between studies may be due to differences in measurement techniques, units used, and the lack of data standardization.

K is involved in numerous essential functions, including transmembrane transport, the activation of glycolytic enzymes, and macromolecule synthesis [28]. It is hypothesized that elevated K levels in the embryo culture medium may contribute to increased blastocyst formation rates and higher cell counts in morula and blastocysts. However, excessively high K concentrations may counteract this beneficial effect [28]. K levels in follicular fluid are thought to play a significant role in the implantation process. Additionally, extremely high K levels, exceeding 405.71 mg/kg, have been associated with polycystic ovary syndrome [29]. In this study, higher K levels were observed in non-pregnant women (0.00564 ± 0.000590 ppm) and those with a follicle diameter of 1–3 cm (0.00563 ± 0.000498 ppm).

A study by Akarsu et al. [30] found that Pb concentrations (22.27 ± 1.73 μg/dl) in the follicular fluids of cows raised near a thermal power plant were higher than those raised in other areas. However, levels of Hg (<0.075 μg/dl), Cd (<1 μg/dl), and radioactivity parameters showed no significant differences. In the present study, we found that the concentration of radioactive elements in the follicular fluid did not vary based on several factors, including the mare’s pregnancy status, the month of pregnancy (if applicable), the presence of a corpus luteum in the ovaries, the diameter of the corpus luteum (if present), the presence of follicles, and the diameter of the largest follicle.

## Conclusion

Despite extensive searches, no previous studies have examined the levels of heavy metals and radioactive elements in the ovarian follicular fluid of pregnant mares, making this publication a pioneering contribution to the field. The data collected in this study suggest that the levels of heavy metals and radioactive elements remain consistent across different physiological conditions in the ovaries of both pregnant and non-pregnant mares. Therefore, it appears that environmental pollution does not significantly impact the follicular fluids in these mares. However, it remains crucial to carefully monitor heavy metals and radioactive elements in follicular fluids to assess the extent of environmental pollution and its potential effects on reproductive health.

## List of Abbreviations

Cd, cadmium; FSH, fllicle-stimulating hormone; Pb, lead; Hg, mercury; Sb, antimony; Co, cobalt; K, potassium; U, uranium; Ni, nickel; Th, thorium.
